# Promoting access equity and improving health care for women, children and people living with HIV/AIDS in Burkina Faso through mHealth

**DOI:** 10.1093/pubmed/fdy196

**Published:** 2018-12-14

**Authors:** M Yé, M Kagoné, A Sié, C Bagagnan, H Sanou, O Millogo, V Duclos, I Tinto, Gilles Bibeau

**Affiliations:** 1 Nouna Health Research Centre, BP 02, Province de la Kossi, Burkina Faso; 2 Assistant Professor, Center for Science, Technology and Society, Department of Global Studies and Modern Languages, Drexel University, Philadelphia, PA 19104, USA; 3 Université de Montréal, CHU Sainte-Justine, Canada

## Abstract

**Background:**

In Burkina Faso, access to health services for women, children and people living with HIV/AIDS (PLWHAs) remains limited. Mobile telephony offers an alternative solution for reaching these individuals. The objective of the study was to improve equity of access to health care and information among women and PLWHAs by reinforcing community participation.

**Methods:**

Using a quasi-experimental approach, a mobile telephone system was set up at five health centres to provide an automated reminder service for health care consultation appointments. Performance evaluations based on key performance indicators were subsequently conducted.

**Results:**

A total of 1501 pregnant women and 301 PLWHAs were registered and received appointment reminders. A 7.34% increase in prenatal coverage, an 84% decrease in loss to follow-up for HIV (*P* < 0.001) and a 31% increase in assisted deliveries in 2016 (*P* < 0.0001) were observed in intervention areas. However, there was no statistically significant difference between intervention site and control site (*P*= 0.451 > 0.05) at post-intervention. Efforts to involve community members in decision-making processes contributed to improved health system governance.

**Conclusion:**

Mhealth may improve maternal and child health and the health of PLWHAs. However, establishment of a mHealth system requires taking into account community dynamics and potential technological challenges.

**Keywords:**

access to care, Burkina Faso, equity, health system governance, mobile telephony, Nouna

## RESUMEN

**Contexto:**

En Burkina Faso el acceso de las mujeres, los niños y las personas con VIH/SIDA (PVVS) a los servicios de salud sigue siendo limitado. La telefonía móvil ofrece una solución alternativa para alcanzar a estos individuos. El objetivo de este estudio fue mejorar la igualdad de acceso a la información y el cuidado de la salud para las mujeres y las PVVS al reforzar la participación comunitaria.

**Métodos:**

Por medio de un enfoque cuasi experimental, se estableció un sistema de telefonía móvil en cinco centros de salud para brindar un servicio de recordatorio automático para las citas de consulta de cuidados de la salud. A continuación, se llevaron a cabo evaluaciones de rendimiento en base a los indicadores de rendimiento clave.

**Resultados:**

Se registró un total de 1.501 mujeres embarazadas y 301 PVVS quienes recibieron recordatorios para las citas. Se observó lo siguiente en las áreas de intervención: un 7,34% de aumento en la cobertura prenatal, un 84% de disminución en la falta de seguimiento para VIH (*P* < 0.001) y un aumento del 31% en partos asistidos en el 2016 (*P* < 0.0001). No obstante, no hubo una diferencia estadística importante entre el sitio de intervención y el sitio de control (*P* = 0.451 > 0.05) en la posintervención. Los esfuerzos por involucrar a los miembros de la comunidad en los procesos de toma de decisiones contribuyeron para una gobernanza mejorada de los sistemas de salud.

**Conclusión:**

Es posible que mSalud mejore la salud materna, la del niño y la salud de las PVVS. No obstante, para establecer un sistema mSalud se necesita tener en cuenta las dinámicas comunitarias y los desafíos tecnológicos potenciales.

**Palabras clave:**

acceso al cuidado, Burkina Faso, igualdad, gobernanza de los sistemas de salud, telefonía móvil, Nouna

## RÉSUMÉ

**Contexte:**

Au Burkina Faso, l’accès aux services de santé pour les femmes, les enfants et les personnes vivant avec le VIH/sida demeure limité. La téléphonie mobile offre une solution de rechange pour joindre ces individus. L’objectif de l’étude consistait à améliorer l’égalité d’accès aux soins de santé et à l’information pour les femmes et les personnes vivant avec le VIH/sida en renforçant la participation communautaire.

**Méthodologie:**

À l’aide d’une approche quasi expérimentale, un système de téléphonie mobile a été installé dans cinq centres de soins de santé afin d’offrir un service de rappel automatisé pour les rendez-vous de consultation en soins de santé. Des évaluations de rendement se fondant sur des indicateurs de rendement clés ont été effectuées par la suite.

**Résultats:**

Au total, 1 501 femmes enceintes et 301 personnes vivant avec le VIH/sida ont été inscrites et ont reçu des rappels pour leurs rendez-vous. Une augmentation de 7,34 % de la couverture prénatale, une diminution de 84 % de l’interruption du suivi pour le VIH (*P* < 0,001) et une augmentation de 31 % des accouchements assistés en 2016 (*P* < 0,0001) ont été observées dans les zones d’intervention. Toutefois, il n’y a pas eu de différence statistiquement significative entre le site d’intervention et le site de contrôle (*P* = 0,451 > 0,05) après l’intervention. Les efforts déployés pour faire participer les membres de la communauté aux processus de prise de décisions ont contribué à améliorer la gouvernance du système de santé.

**Conclusion:**

La santé mobile peut améliorer la santé des femmes enceintes, des enfants et des personnes vivant avec le VIH/sida. Cependant, l’établissement d’un système de santé mobile nécessite de prendre en compte la dynamique communautaire et les défis technologiques potentiels.

**Mots-clés:**

accès aux soins, Burkina Faso, égalité, gouvernance du système de santé, téléphonie mobile, Nouna

## Background

Maternal, neonatal and child mortality rates in developing countries remain high and represent the main source of inequity in public health.^1^ In the context of poverty, low education, high maternal and child mortality rates in Africa, the New Partnership for Africa’s Development in 2002 identified launching a campaign to improve health through use of new information and communication technologies (ICTs) as one of its 10 main priorities.^2^ The initiation of pilot projects began soon afterwards in the member countries of Senegal, Rwanda, Mauritania and Mali.^3^

ICTs appear to offer potential benefits for disseminating health information among the population. According to the secretary-general of the International Telecommunications Union, ICTs make the world a much better place, particularly for the most disadvantaged, including women, youth and persons with handicaps.^4^ Mobile technology can generally be used to overcome geographic constraints and improve communication^5^ ICTs enable real-time medical diagnosis in marginalized rural areas where health services are frequently non-existent.^6,7^ The deployment of mobile devices is also decisive in disease prevention and containment by promoting health behaviour changes among populations.^8^

Leveraging the potential of mobile telephony in improving global health outcomes remains the objective of a number of pilot projects.

One recent systematic review concluded that the provision of appointment reminders via mobile telephone in the form of SMS messages is as effective as voice telephone calls in improving the use of medical services.^9^

A second systematic review by Betjeman *et al*.^10^ confirmed that mHealth may improve and reduce the cost of patient monitoring, treatment compliance and communication among health workers.

With regard to mHealth use in improving PLWHAs’ access to services, several studies have depicted the state of the evidence. A systematic review by Catalani^11^ concluded that there is evidence that mHealth tools can improve linkage to care, retention in care and adherence to antiretroviral treatment (ART). The authors also found that future mHealth efforts should address additional programmatic needs such as voluntary counselling testing (VCT) and prevention of mother to child transmission of HIV (PMTCT).

Kunutor *et al*.^12^ in a pilot study assessed the feasibility and program outputs associated with sending simple text messages or making a voice call to remind PLWHAs patients who miss their appointments in rural Uganda. The study demonstrated that most patients who received text/voice messages presented for treatment within a few days, however, the lack of comparison group makes it difficult to conclude.

In Kenya, Lester *et al.*^13^ tested the effects of sending SMS on ART adherence through a randomized clinical trial (RCT) of HIV-infected adults initiating ART in three clinics. Patients who received SMS support had significantly improved ART adherence compared with the controls.

In a Cochrane systematic review, Horvath *et al.*^14^ searched in a RCTs in which patients receiving ART were provided with mobile phone text messages as a means of promoting adherence to ART. They concluded that there is high-quality evidence from two RCTs that mobile phone text-messaging at weekly intervals is efficacious in enhancing adherence to ART. mHealth also makes it easier to find lost patients^15^ and remains a practical strategy for disseminating and improving patient care in rural areas.^16^

However, for Car *et al*.,^9^ while mobile phone messaging applications could provide an important, inexpensive delivery medium for reminders, there is limited evidence on the effects of mobile phone text message reminders for appointment attendance. This limitation was raised by Aradan^17^ for whom most projects on mHealth were pilot studies or RCT studies implemented at community levels and not yet scaled-up to larger levels, thus, evidence presented on effectiveness is limited and long-term results are unclear.

The current study was conducted in Burkina Faso, where maternal and child mortality remains high (341 maternal deaths per 100 000 live births and 28 neonatal deaths per 1000 live births).^18^ This is far away from achieving the sustainable development goal (SDG) of reducing the global maternal mortality rate (MMR) to <70 per 100 000 live births by 2030.^19^ Persisting HIV/AIDS epidemic (prevalence rate of 1.2%) and a high rate of loss to follow-up among PLWHAs (10%) represent ongoing barriers to an effective response to the epidemic.^20^

The aim of this study was to describe how a mHealth project contributed to improving equity of access to health care for women, children and PLWHAs.

## Methods

### Study setting

The study was conducted in Nouna Health District (NHD) in Burkina Faso. Nouna has hada Health and Demographic Surveillance system (HDSS) data gathering since 1984.^21^ Longitudinal data collection is being done in 25 primary health centres (HCs). The HDSS served as a framework for selection of the HCs.

**Fig. 1:**
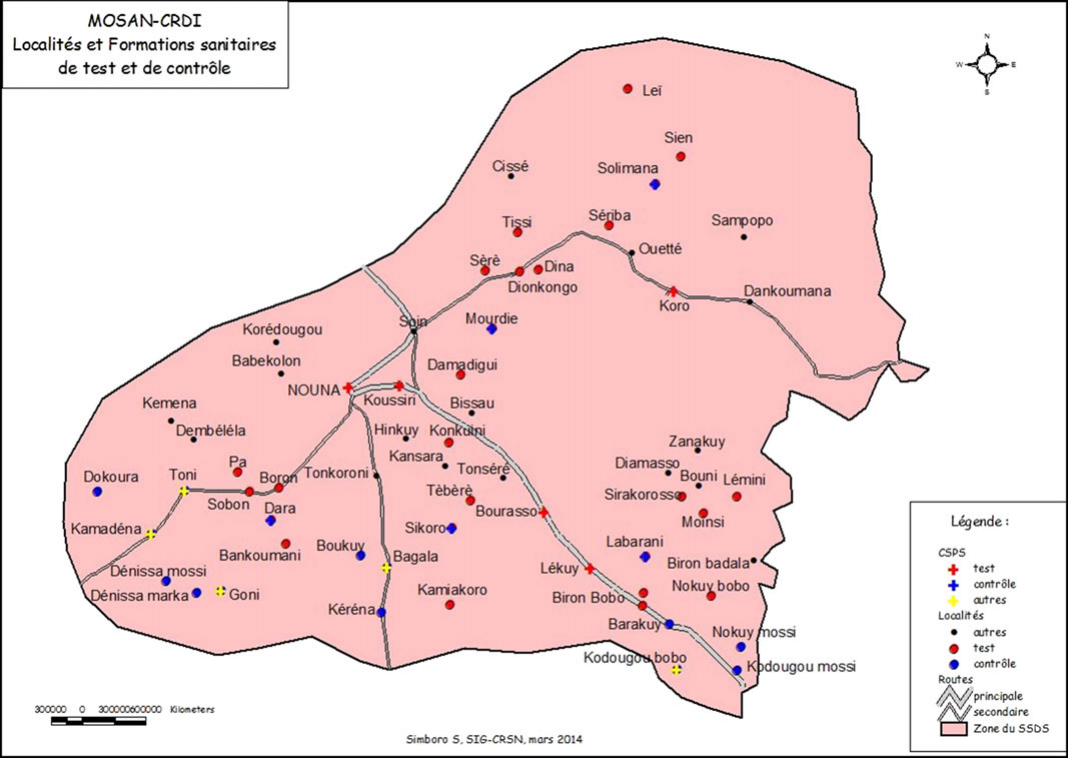
Nouna mHealth project area within the HDSS. HDSS, Health and Demographic Surveillance System.

Figure 1 illustrates the study area.

### Description of mHealth intervention

The intervention involved equipping 52 godmothers and 10 HIV/AIDS facilitators—with free mobile telephones. Godmothers are former traditional birth attendants used now in HCs to conduct community-based activities to support health care provision (sensitization, home visits, counselling of pregnant women, etc.). HIV/AIDS facilitators are members of registered associations working with health services to support HIV/AIDS patients care. Their activities focusses on PLWHAs to increase their adherence to service, compliance with treatment and retrieval of patients lost to follow-up.

#### Description of mHealth technological platform

The project mHealth description covers only some relevant criteria of the mHealth evidence reporting and assessment checklist (mERA).^22^

### Description of software, hardware and physical infrastructure

The MOS@N project was developed using the open-source Java programming language relying on J2EE JSP/Servlet technology and standard web services technologies.

MOS@N features an interactive voice response (IVR) and SMS transmission system functioning in five local languages (French, Dioula, Moore, Dafing and Bwamu). The mHealth system was programmed to send automated reminders to Godmother and HIV/AIDS facilitator’s phone via SMS at regular intervals once every day. Five IVR modules for patients management were developed, namely: (i) IVR module to deliver awareness and sensitization messages to patients; (ii) IVR module for patient’s data management and follow-up; (iii) IVR module for appointment or return visit reminders; (iv) IVR module for transferring calls to the caller’s referral HC; and (v) IVR module for calls transfer to referral HCs.

**Fig. 2:**
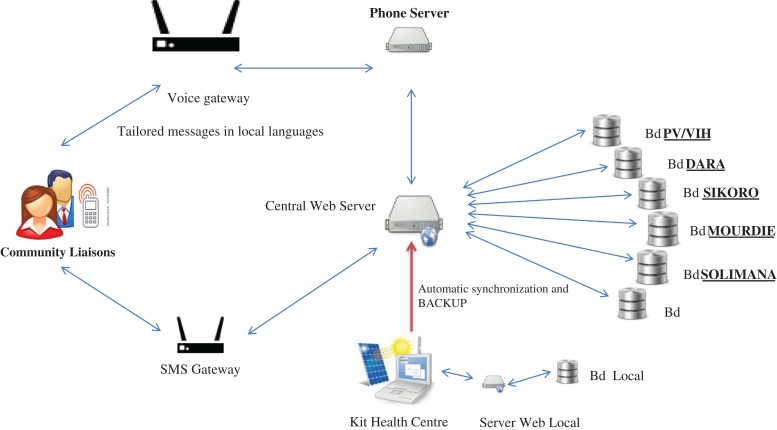
mHealth platform structure.

The system architecture is based on five components: a mobile Internet connection, a Web server to host the database and application, a main database, a communication server with voice mail and email functionality that interacts with the server and peripheral system (mobile telephones and the computers based at HCs).^23^ Figure 2 depicts the conceptual model of the platform.

#### Description of mHealth enabling environment at population level

Within the HDSS, the mobile phone possession rate increased from 3.8% in 2006 to 63.80% in 2013. This growth is consistent with that reported at national level: ~66%.^24^

In addition, HCs capacity assessment was conducted prior to the intervention, and deficiency in power supply was addressed by solar panel deployment at health facility level coupled with provision of mobile energy kits to community health workers involved in the project for mobile phone charging in addition to regular communication credit allowance (10 $/month).

### Study design and type

This was a cross-sectional study of a community-based mHealth project using quasi-experimental design. Five intervention and five control sites out of 17 HDSS HCs, were selected to participate in the study.

#### Sampling methods

Firstly, convenience sampling was used to selected HCs that met some criteria (two health staff, a maternity ward, a dispensary, functioning solar panel system, and accessibility to health district). The 17 HCs out of 25 met that criteria. Secondly, a simple random sampling was used to select five HCs for intervention and five for control out of the 17 HDSS sites. For HIV/AIDS intervention, the sampling base was the whole district with regard to the centralized HIV/AIDS database at district level.

For qualitative study, convenience sampling was used to enrol participants until the required sample size is reached.

#### Data collection methods and tools

A structured questionnaire was used to record information on key statistics at HCs and district level in 2013 and 2016. In addition, the degree of acceptance of mobile telephony was explored among 52 godmothers, 10 HIV/AIDS facilitators and 15 health workers, using the standard technology assessment model (TAM).^26^

Qualitative approach was use to collect data among key respondents. Individual in-depth interview and focus group discussions (FGDs) were favoured: 10 FGDs with 91 women, while 35 semi-structured individual interview were conducted with (10 health workers (HWs), 10 community health workers (CHWs), 5 godmother and 2 HIV/AIDS facilitators).

#### Data analysis

Quantitative data analysis was performed by STATA.11. A *t*-test for two independents samples means was used to estimate the difference at pre- and post-intervention for key indicators prenatal care (PNC1), prenatal care 4(PNC4), tetanus toxoid (TV2+), intermittent treatment of malaria (IPT2), high pregnancy referred (HRP), prevention of mother to child HIV (PMTCT), assisted deliveries (AD,) postpartum care (PPC) and polio and BCG, lost to follow-up of HIV/AIDS patients. The results were significant if *P*-value <0.05.

For qualitative data analysis, recorded interviews were transcribed in French and imported into the application ATLAS.ti4.2. The content analysis method was used, with a focus on the manifest content and treating all statements in the verbatim transcriptions as complete units.^25^

#### Ethical consideration

The study was approved by Burkina Ethical Committee for Health Research (CERS/2013-12-115). Prior to interview, all participants’ voluntary consent was sought either written or verbal.

## Results

### Acceptance of mHealth project

In general, 100% of interviewee’s participants recognized the important role that telephone can play as a tool for disseminating information.

The godmothers were knowledgeable to use the mobile telephones to reach others—such as their husbands—in a problem situation and that it can help to reduce both distances and health care costs. With respect to the acceptance of mobile telephony gauged by TAM described by Davis.^26^ The perceived usefulness of the mobile telephone was 94% among godmothers, 90% among HIV/AIDS facilitators. About 74% of health workers were entirely in agreement with using mobile telephony to transmit health information and contact patients. Mobile phone was also perceived at effective mean to deal with emergency situation as pointed out by a health worker.

### Results related to mHealth intervention 30 months post-implementation

#### Assessment of implementation process and recordings made of by Godmothers and HIV/AIDS facilitators

To follow up activities carried out involving the godmothers and HIV/AIDS facilitators, a reporting system was established to summarize the work performed. A total of 12 756 alerts were received by Godmothers and HIV/AIDS facilitators, and 1501 pregnant women were referred to HCs.

Over the same period, information on more than 1237 of the 6272 pregnant women attending prenatal care visits (PNC1 or PNC2) was recorded on the mHealth platform via the HCs.

#### Recording of PLWHAs

The list of PLWHAs was kept in a centralized database confidentially. A total of 301 patients were registered at HIV referral hospital in 2013 prior to the intervention and 1008 patients were in the database as of 31 December 2016. A significant decrease is noted in the rate of PLWHAs lost to follow-up, which dropped by more than 84% from 10% (32 lost to follow-up out of 301 followed) in 2013 to <1.6% (17 out of 1008) in 2016 (*P* < 0.0001).

### Level of achievement of indicators in intervention area between 2013 and 2016

The intervention area saw an increase in certain key indicators from baseline 2013 to post-intervention 2016. For example, PNC1 visits increased by 117% (*P* < 0.0001), the proportion of pregnant women receiving TV2+ increased by 43.46% (*P* < 0.0001), and the proportion of AD by 30.77% (*P* < 0.0001).

Table 1 summarizes the performance levels attained between 2013 and 2016.

**Table 1:** Evolution of indicators in intervention area between 2013 and 2016

Indicators	2013 (%) (A)	2016 (%) (B)	Difference 2016–13 (C)	Progress (%) (D)**	*P*-value
PNC1	40 (349/873)	86.77 (931/1073)	46.75	117	<0.0001
PNC4	52.8 (461/873)	56.66 (608/1073)	3.88	7.34	0.088
TV2+	62 (543/873)	89.00 (955/1073)	26.95	43.46	<0.0001
IPT2	85 (743/873)	83.69 (898/1073)	−1.26	0	0.42
HRP	56 (18/32)	100 (61/61)	44	78.57	<0.0001
PMTCT	78.33 (684/873)	85.27 (915/1073)	6.93	8.84	0.0001
AD	62.5 (446/713)	82.07 (760/926)	19.54	30.77	<0.0001
PPC	62 (429/691)	54.99 (380/691)	7	11.29	0.08
CP	50.8 (444/873)	43.43 (76/175)	−6.96	0	0.07
Polio birth	100 (586/586)	100 (716/716)	0	0	>0.05
BCG	100 (586/586)	100 (716/716)	0	0	>0.05
Combined	68.18	80.18			0.85

PNC1, prenatal consultation 1; PNC4, prenatal consultation 4; TV2+, tetanus vaccine, two or more doses; IPT2, intermittent preventive treatment for malaria in pregnant women second dose; HRP, referred high-risk pregnancies; PMTCT, prevention of mother-to-child transmission of HIV; AD, assisted delivery; PPC, postpartum consultation; CP, contraceptive prevalence; BCG, Bacillus Calmette-Guerin.

**Level of progress (D) = (B−C/) A*100.

### Level of achievement of indicators in control area between 2013 and 2016

From baseline 2013 to post-intervention 2016, there was an increase in PNC1 by 68% but less than that observed in intervention site. There was also an increase by 60% for PPC (*P* < 0.0001). Although some slight progress, the difference between baseline and post-intervention was not statistically significant (*P* = 0.429 > 0.05) as in Table 2.

**Table 2:** Evolution of indicators in control area between 2013 and 2016

Indicateurs	2013 (A)	2016 (B)	Difference (C)	Level of progress (D)**	*P*-value
PNC1	45.09 (410/911)	75.93 (743/978)	30.84	68.40	<0.0001
PNC4	54.87 (499/911)	45.47 (445/978)	−9.4	−17.13	<0.0001
TV2+	87.15 (794/911)	72.59 (778/978)	−7.65	−8.78	<0.0001
IPT2	92.9 (846/911)	95.60 (710/978)	−20.31	−21.86	0.011
HRP	68.75 (11/16)	100 (9/9)	31.25	45%	<0.0001
PMTCT	93.62 (853/911)	83.59 (818/978)	−10.03	−10.71	<0.0001
AD	93.37 (710/761)	89.97 (734/816)	−3.4	−3.64	0.0086
PPC	40.23 (271/674)	64.36 (466/723)	24.13	59.98	<0.0001
CP	74.86 (261/349)	65.93 (246/374)	−9.09	−12.14	0.008
Polio birth	85.86 (662/771)	89.08 (737/827)	3.22	3,75	0.051
BCG	85.86 (662/771)	89.08 (737/827)	3.22	3.75	0.051
Combined	M1 = 77.70	M2 = 79.24			0.429

### Level of achievement of key indicators in intervention area compared to control area

Increases were observed in the intervention area versus the control area, specifically a 10.84% difference in PNC1 coverage (*P* < 0.0001), 11.19% for PNC4 coverage, 9.50% for TV2+ and 10.92% for polio vaccine at birth and BCG (*P* < 0.0001).

However, no change was observed for AD (−7.9% difference), PPC (−9.37% difference) and CP (−22.5% difference). The difference between intervention and control sites was not significant (*P* = 0451), as shown in Table 3.

**Table 3:** Comparison of averages for the test and control areas at the end of December 2016

Indicators	Test area 2016 (%)	Control area 2016 (%)	Difference (%)	*P*-value
PNC1	86.77 (931/1073)	75.93 (743/978)	10.84	<0.0001
PNC4	56.66 (608/1073)	45.47 (445/978)	11.19	<0.0001
TV2+	89.00 (955/1073)	72.59 (778/978)	9.5	<0.0001
IPT2	83.69 (898/1073)	95.60 (710/978)	11.1	<0.0001
HRP	100 (61/61)	100 (9/9)	0	>0.05
PMTCT	85.27 (915/1073)	83.59 (818/978)	1.68	0.427
AD	82.07 (760/926)	89.97 (734/816)	−7.9	<0.0001
PPC	54.99 (380/691)	64.36 (466/723)	−9.37	0.0003
CP	43.43 (76/175)	65.93 (246/374)	−22.5	<0.0001
Polio birth	100 (716/716)	89.08 (737/827)	10.92	<0.0001
BCG	100 (716/716)	89.08 (737/827)	10.92	<0.0001
Combined mean	M1 = 80.16	M2 = 79.22		0.451 (*P* > 0.05)

PNC1, prenatal consultation 1; PNC4, prenatal consultation 4; TV2+, tetanus vaccine, two or more doses; IPT2, intermittent preventive treatment for malaria in pregnant women second dose; HRP, referred high-risk pregnancies; PMTCT, prevention of mother-to-child transmission of HIV; AD, assisted delivery; PPC, postpartum consultation; CP, contraceptive prevalence; BCG, Bacillus Calmette-Guerin.

Similarly, baseline comparison between intervention and control sites in 2013 did not show any statistically significant difference (combined mean *P* = 0.221), as shown in Table 4.

**Table 4:** Baseline comparison between intervention and control areas in 2013

Indicators	Test area 2013 (%)	Control area 2013 (%)	Difference test area–control area 2013	*P*-value
PNC1	40 (349/873)	45.09 (410/911)	−5.09	0.032
PNC4	52.8 (461/873)	54.87 (499/911)	−2.07	0.38
TV2+	62 (543/873)	87.15 (794/911)	−25.15	<0.0001
IPT2	85 (743/873)	92.9 (846/911)	−7.9	<0.0001
HRP	56 (18/32)	68.75 (11/16)	−12.75	0.399
PMTCT	78.33 (684/873)	93.62 (853/911)	−15.29	<0.0001
AD	62.5 (446/713)	93.37 (710/761)	−30.87	<0.0001
PPC	62 (429/691)	40.23 (271/674)	21.77	<0.0001
CP	50.8 (444/873)	74.86 (261/349)	−24.06	<0.0001
Polio birth	100 (586/586)	85.86 (662/771)	5.09	<0.0001
BCG	100 (586/586)	85.86 (662/771)	5.09	<0.0001
Combined mean	M1 = 68.18	M1 = 74.77		0.221

### Assessment of equity of access to care and participation to health governance

Equity of care presumed the removal of any barriers potentially preventing access to services due to a person’s social, economic or geographic status. In this regard, the government provided free health services to children under the age of five and pregnant women.

In our study, ~96% of women attending PNC visits in intervention area and 88% in control area were not aware of the availability of free care. The difference between the intervention and control sites was not statistically significant (*P* = 0.0739). Across all sites, there was limited local community participation in health system governance in terms of involvement in decision-making processes.

### Challenges to implementation of the technological platform

Challenges related to technology arose in relation to the availability of rugged mobile telephones appropriate for use in a rural environment and extreme weather, with more than 65% of telephones being damaged and replaced. Poor availability of connectivity was also noted. Ideally, HCs would communicate with the main server via the 3 G network, but in reality, telephone service was very poor to non-existent in the intervention area.^27^ Alternative solutions were proposed, such as copying data onto a USB flash drive to update the server.^28^

Additionally, some community members had difficulty using their telephones, preventing effective use of the devices. Despite relatively good availability of mobile telephones in the study area (60%), the majority of community members found themselves using a telephone for the first time. Training of the community members (Godmothers and HIV/AIDS facilitators) took longer than anticipated, due in part to the fact that 90% of them (56 out of 62) could not read or write. With respect to health workers, only 35% were familiar using software on computers. This created a need for a series of training sessions before the start of the project.

Human resource challenges took the form of a high turnover among the trainees (6 out of 20), which was beyond our control.

## Discussion

### Main findings of this study

Mobile phones contribute to closing the communication gap between various population groups, notably between the rich and the poor. After 30 months of implementation, the MOS@N project helped improve certain indicators, such as prenatal care coverage and assisted deliveries. Although studies examining the impact of mHealth in developing countries have remained relatively limited,^29^ the few conclusions of studies similar to our own have shown that the rapid proliferation of mobile telephony provides an opportunity for the taking when it comes to improving health care and services in developing countries.^29,30^

### What is the evidence to date on this topic?

Mhealth demonstrated its effectiveness in contributing to improving patient health in relation to both mother–child health and PLWHAs. Several other studies besides our own have clearly shown the role of ICTs in improving health indicators. One such study, Weltel Kenya1, conducted in Kenya, showed that patients who are sent reminder messages about taking medications are more compliant than those who are not.^13^ Another pilot project implemented in Uganda, Childhood ART Adherence (CHARTA), used IVR servers and SMS messages to improve compliance among a cohort of 121 children living with HIV/AIDS in Mbarara.^31^ Studies have also suggested that equipping community liaisons with mobile telephones remains a practical strategy for finding patients lost to follow-up^15^ and delivering care to patients in rural areas.^16^ Despite this encouraging context, the majority of successful mHealth programs in limited-resource environments face certain challenges.^32^ As indicated in the present study, the problems encountered include poor or non-existent telephone service^31^ and high cost of Internet access and lack of electricity.^32–35^ Additionally, illiteracy and the use of vernacular languages pose problems when it comes to understanding messages.^31,36^

With regard to PLWHAs, improved follow-up is noted, with a decrease of more than 84% in the proportion lost to follow-up. However, documentation of experiences of PLWHA follow-up via mHealth has been very limited in the literature, doubtless due to the sensitive nature of the disease and the precautions required upstream in the system to maintain confidentiality.

Nevertheless, one randomized study in Kenya aiming to demonstrate the role of mHealth technology in compliance with ARV treatment revealed more than 90% compliance during the first 3 months of the study in the intervention group versus 40% in the control group.^37^

In Uganda, a similar randomized study of two groups of 63 ARV patients followed for 3 months showed that the use of SMS reminders contributed to achieving more than 97% compliance in the intervention group, while compliance was only 11% in the control group.^38^ Although the sample sizes in these studies do not permit generalization, the studies did demonstrate on a small-scale the feasibility of mHealth in the context of PLWHA follow-up and its potential role in improving treatment compliance and attendance at follow-up appointments.

### What this study adds to the knowledge

Based on the results observed under the MOS@N project, the introduction of a mobile telephony intervention appears to generate positive changes in critical health indicators. The present study, like others, demonstrates how SMS and voice messages for appointment reminders can contribute to improving equity of access to care. These are contingents on responding to the local social and cultural realities, and anticipating challenges related to technology deployment. These approaches include addressing both technological aspects (adaptation of telephones to host environment) and human aspects (gender, ability to utilize the communication system in place). In the absence of effective follow-up and coordination along these technological and human dimensions, mHealth interventions are prone to failure.

The role of MOS@N in improving mother–child and PWLHAs health indicators generates hope for the possible large-scale implementation of the mHealth technology in low-income and developed countries.

### Study limitations

The study was conducted at five sites in NHDSS out of the 17 HCs, limiting the study’s representativeness. With regard data collection, in-depth interviews were conducted in local language and then translated back into French, creating a risk of distortion of participant’s opinions.

Lastly, the comparison was not possible for lost to follow-up for HIV/AIDS patients between intervention and control groups. This could have introduced an interpretation bias due to use of single district level data.

## Conclusion

An intervention research study based on use of mHealth showed a high-level of acceptance among providers and beneficiaries. The project contributed to improving access of pregnant women, children and PLWHAs to directed care that meets their expectations.

Increases in certain indicators are evidence that the project contributed to strengthening health service delivery. The emphasis on working with community members was shown to improve participation and promote more inclusive governance of health system. However, it is important to anticipate the technological and human challenges associated with the use of mHealth in these contexts.

## Supplementary Material

Supplementary Data

Supplementary Data
