# Supplementary material for: Promoting access equity and improving health care for women, children and people living with HIV/AIDS in Burkina Faso through mHealth
Source: J Public Health (Oxf). 2018 Dec 14;40(Suppl 2):ii42–51. doi: 10.1093/pubmed/fdy196 (PMC6294034; doi:10.1093/pubmed/fdy196)
Supplement: Supplementary Data [file fdy196_supplementary_file_1_description_du_projet_mosan_mera.docx]

***Description of the mHealth intervention using mERA guideline***

The mHealth project (MOS@N) is an initiative of the Nouna Health Research Center supported by IDRC/Canada in 2013. It fall into a global integrated approach to use information and communication technologies (ICTs), in particular the mobile phone, which has a good penetration rate in local setting.

MOS@N is an innovative emerging local project that involved ICTs technicians, health workers, godmothers, social scientists, and funders, including IDRC/Canada.

The project interconnected five primary health centre of Nouna Health District through IT infrastructure tailored in the needs and participation of community and funded by IDRC/Canada over three years period from 2013 to 2016.

The other stakeholders involved in the project implementation are Burkina Faso’s Ministry of Health Research Directorate, and the Global Health Program of the McGill University's Faculty of Medicine/Canada and University of Montreal.

Mos@N has been implemented as part of the IDRC health programme on equity-health-governance and health systems strengthening through ICTs program aimed at improving coverage of maternal and child care and People Living with HIV/AIDS (PLHAV).

1. **Infrastructure(population level)**

Indeed, in the Health and Demographic Surveillance site of Nouna (HDSS), the mobile phone possession rate increased from 3.8% in 2006 to 63.80% in 2013. This growth is consistent with that reported a national level about 66% (ARCEP, 2015).

In terms of communication coverage, the intervention zones were covered by national three main phone ad internet companies with acceptable network quality and internet bandwidth.

The fact that mobile network coverage stands at 95%, and more than 66% of the population subscribes to one of the domestic mobile networks country-wide, were enabling environment for adoption of mHealth solutions at community level for health service delivery. In addition, health facility capacity assessment was conducted prior to the intervention, and deficiency in power supply was addressed by solar panel deployment at health facility level coupled with provision of mobile energy kits to community health workers involved in the project for mobile phone charging in addition to regular communication credit allowance (10US/month).

**2. Technology platform**

The technology platform is an interactive communication computer system based on mobile technologies from a local provider. The platform was developed in Java language. It relies on the J2EE JSP / Servlet technology framework and standard web services technologies to interact with external elements.

The platform is built around a system includes the recording of patient data at health facility level through electronic register inter-connected within a networks of computer distributed within five health facilities. In addition, the platform with internet access has a feature to send automatic message to godmothers cells-phone preprogrammed with users registered in the system. The system allow also sending reminder messages to appointment visits supported by a synchronizing databases hosted and managed by central server at the Nouna Health research centre supervised by IT manager.

All community relays about sixty (60) composed by godmothers, interface with antenatal care services and members from HIV/AIDS association are equipped with cell-phones to all serve as an interface between the beneficiaries and the health services in the transmission of information automatically sent on their telephone. The interactive voice messaging system (IVR) was developed and incorporated five local languages (Bwamu, Dafing, Dioula, Mooré and French) to overcome literacy barriers. In addition, an automated patient reminder system for follow-up appointments was incorporated in the technology that was developed by local experts familiar with mobile health technology. For ease of deployment, open software developed under PHP Symphony Framework was adopted.


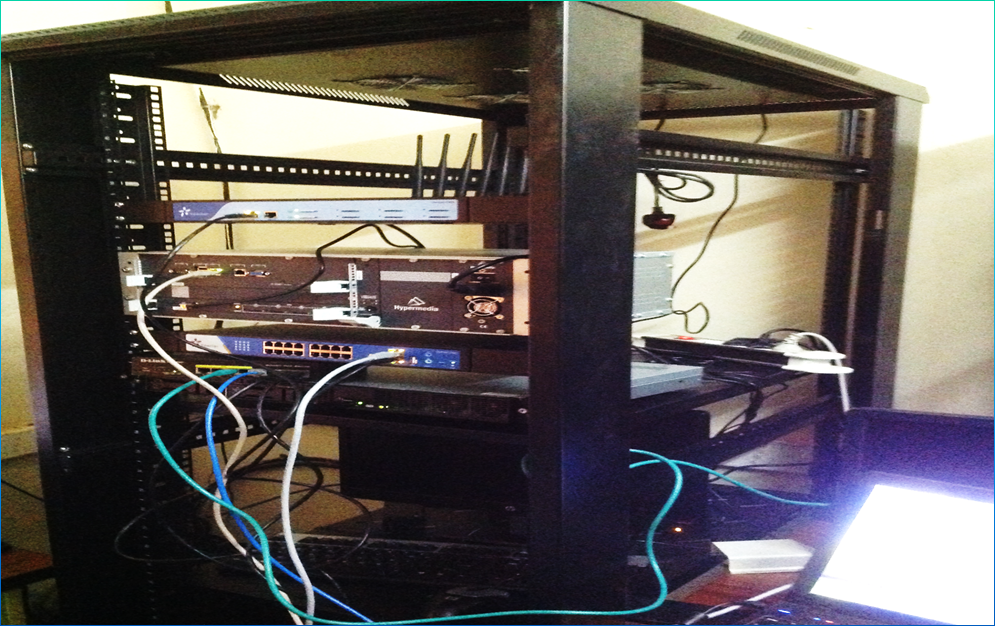


**Picture of physical infrastructure**

***3.Type of mHealth equipment used***

The equipment used consists of an HP ProLiant DL320e Gen 8 v2 server, a Cisco 2800series router, a Hypermedia SMS gateway, a YeaStar U100 telephone server (PABX) and a Neogate TG 800 voice gateway, all managed by a local telecommunication operator.

The mHealth technology incorporated the following components: a GSM internet connexion, an Interactive Voice Messaging System, an automatic patient reminder system, and an automated backup system including data synchronization with a central server hosted in Nouna. In addition, a core health information system was developed and incorporated within the district health facilities to collect data relevant for decision-making.

***4.Intervention content/delivery***

Five modules were developed for the overall content of the system through interactive voice and response system (IVR):

- IVR for patient management, which was developed to deliver awareness and sensitization messages but also manage local access to the content hosted at the central server,
- IVR to manage patients ’data which allow data saving and follow-up
- IVR for appointment or planned visits dates reminders. This was developed to enable reminders alert sending based on pre-programmed visits dates at health facility level. This component has also a feature to validate each visit performed by community member toward beneficiary.
- IVR for transferring calls to the caller’s referral health centre. This software allows to transfer the alerts of the godmothers and animators to the appropriate HC. The request is therefore received and processed by respective in-charge either godmother or association of PLWHA.
- IVR for calls transfer to referral health centre. This allow to transfer alerts from godmothers or member of PLWHA to appropriate health centre. The health facility then receives and proceed with information analysis.

**5. Patient management system**

The patient management system put in place allows for the recording of patient informations and schedule visits for pregnant women, postpartum women, PLWHA, and also newborn vaccination. This system has been configured to synchronize the daily data recorded at HC platform with the central database located at Nouna Health Research centre server.
The patient management system was developed with PhP symfony2 framework and a MySQL database. It has been deployed in an Apache2 server. Daily backups are performed by Windows tasks that run the MySQLdump program.
Several tests were conducted with the project team prior to system deployment. A technology evaluation was conducted by an IT solution expert "Development - Audit -Informatics - Personalized Monitoring.

**6. IVR to recall visits dates**

This IVR sends appointment reminders ay Day-5, Day-3, and Day-1 prior to the visit date. In case the patient does not show up for the visit (eg prenatal consultation), another Day + 1 reminder, after the visit date, is sent. The implementation of this system was entrusted to a consultant, expert in the field, supported by an officer from CRSN. After identification of the voice messages to be delivered to the sponsor or the facilitator, the CRSN recorded these messages.

For voice system design, an asterisk server was used. This asterisk server is a software that contains all the tools and defines the mechanisms for setting up a voice interaction server.

The mobile phone provided to godmothers and HIV/AIDS facilitators were configured with shortcut keyboards.
