# Supplementary material for: Promoting access equity and improving health care for women, children and people living with HIV/AIDS in Burkina Faso through mHealth
Source: J Public Health (Oxf). 2018 Dec 14;40(Suppl 2):ii42–51. doi: 10.1093/pubmed/fdy196 (PMC6294034; doi:10.1093/pubmed/fdy196)
Supplement: Supplementary Data [file fdy196_supplementary_file_2_evaluation_technologique_plateforme_mosan.docx]

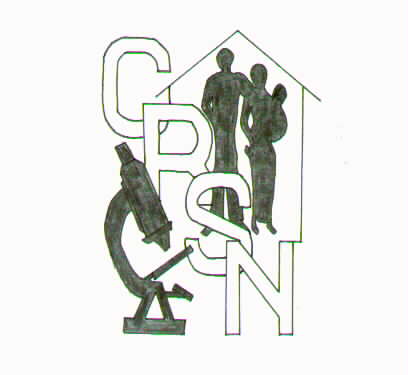


| **Centre de Recherche en Santé de Nouna (CRSN)**  **PROJET MOS@N**  **Rapport de l’évaluation technologique du projet MOS@N** |
| --- |

# INTRODUCTION

## Contexte et objectifs du projet MOS@N

Le Centre de Recherche en Santé de Nouna (CRSN) en partenariat avec l’Hôpital Sainte Justine de Montréal au Canada a obtenu de façon compétitive un financement du Centre de Recherches International pour le Développement (CRDI/CANADA) pour la mise en œuvre d’un projet de recherche intitulé « Amélioration de la gouvernance et de l’équité par l’utilisation des Technologies de l’Information et de la Communication (TIC) : Cas pratique appliqué aux soins de santé maternelle et infantile, et à la prise en charge des personnes vivant avec le VIH (PV/VIH) dans le District sanitaire de Nouna » couvrant la période 2013-2015.

Ce projet de recherche-intervention est mis en œuvre conjointement avec le District sanitaire de Nouna et vise les objectifs suivants :

#### Objectif général

Contribuer à l’amélioration de la couverture des soins maternels et infantiles, et des PV/VIH, à travers l’utilisation de téléphones mobile en milieu rural, de manière à assurer une meilleure gouvernance et équité d’accès aux soins dans le District sanitaire de Nouna, au Burkina Faso.

#### Objectifs spécifiques

- Mettre en place une plate-forme santé-mobile dans 5 CSPS du DSN qui permettra d’intégrer le Système de surveillance démographique et de santé et d’autres systèmes d’information sanitaires (SIS),
- Renforcer les capacités de l’équipe de recherche, ainsi que des acteurs des soins maternels et infantiles et des services de PVVIH en matière de recherche, d’analyse et d’utilisation des données provenant du SIS (incluant la plate-forme santé-mobile),
- Evaluer l’acceptabilité du système de santé mobile par les marraines, les animatrices, les bénéficiaires et les prestataires de soins et la façon dont l’utilisation du système contribue à des changements dans la prise de décision, la participation des individus (y compris ceux qui sont les plus vulnérables), l’accès et l’utilisation des services, et d’autres questions liées à la gouvernance et à l’équité,
- Conduire une évaluation cout-efficacité et équité de l’intervention afin d’informer la meilleure façon d’intégrer la plate-forme de santé-mobile dans le système de santé général,
- Assurer une large diffusion des résultats significatifs du projet,
- Identifier le potentiel de généralisation du projet à l’échelle national.

## Présentation du présent rapport

Le présent rapport comporte trois (3) grandes parties qui sont:

- Une synthèse des Interviews,
- Une étude et évaluation de la plateforme technologique,
- Les orientations et les recommandations.

# Synthèse des Interviews

## Centre de Dépistage et de pris en charge Médicale du VIH/SIDA(CDPM)

| **Organisme** : Projet MOS@N | |
| --- | --- |
| **Code** : 01  **Date**: 29/04/2015 | Interviewé : Ouédraogo Tiguéni |
|  |  |
| **Niveau de la maitrise du logiciel** : Bonne maitrise  **Description du poste** : CDPM (Centre de Dépistage et de prise en charge Médicale)  **Tâches du Poste :**   - Sensibiliser des patients VIH, - Dépister et suivre les patients VIH, - Enregistrer les personnes vivantes avec le VIH, - Effectuer les examens biologiques et traitement ARV des patients, - Effectuer et enregistrer les consultations réalisées.   **Les difficultés liées au logiciel** :   - Les Rendez-vous sont programmés mais les rappels par messages vocaux ne sont pas effectifs (5^e^ jour- 3^e^ jour- la veille), - Des messages d’erreurs liés au serveur web qui ne démarre pas automatiquement, - Impossibilités d’ajout direct d’une localité lors de l’enregistrement des personnes hors zones (ce cas concerne les patients venant d’autres localités), - Le champ « **Age** »n’est pas contrôlédans le formulaire d’enregistrement des patients c'est-à-dire possibilité d’obtenir des âges sur 3 chiffres (exemple: 500 ou 1000), - Un problème lié à la navigation dans le logiciel exemple : la validation du formulaire de modification ne renvoie aucun message, le même formulaire est affiché,   **Les équipements :**   - Un ordinateur portable HP Probook 4540 s. | |

## CSPS de DARA

| **Organisme** : Projet MOS@N | |
| --- | --- |
| **Code** : 02  **Date**: 30/04/2015 | Interviewé : Mme |
|  |  |
| **Localité** : DARA  **Niveau de la maitrise du logiciel** : Insuffisant  **Description du poste** : CSPS (Maternité)  **Nombre de marraines** : 16 marraines couvrent le CSPS  **Tâches du Poste :**   - Dépistage des grossesses et enregistrement des femmes enceintes, - Programmation et enregistrement des consultations prénatales des femmes enceintes, - Programmation et enregistrement des consultations postnatales, - Enregistrement des naissances et suivi des nouveau-nés.   **Les Difficultés liées au logiciel** :   - Dans le formulaire d’enregistrement, le champ « **Age de la grossesse »**n’est pas contrôlé ; il doit être compris entre 6 et 45 semaines, - Les messages d’erreursde contrôle des champs sont en anglais rendant la compréhension difficile pour les utilisateurs, - L’âge n’apparaît pas dans le formulaire de modification de patiente, - Leformat de saisie de valeurs de champs diffère suivant l’utilisateur (Exemple : « 10 kg »diffèrede« 10KG » diffère de« 10 Kg »), - Un problème de navigation dans l’application : la validation de certains formulaires ne renvoie aucun message.   **Les équipements :**   - Un ordinateur portable HP Probook 4540s, - Deux accumulateurs d’énergie de 100A/H et un convertisseur de tension (24v), - Une plaque solaire de 250 Watts. | |

##

## CSPS de SIKORO

| **Organisme** : Projet MOS@N | |
| --- | --- |
| **Code** : 03  **Date**: 30/04/2015 | Interviewé : |
|  |  |
| **Localité** : SIKORO  **Niveau de la maitrise du logiciel** : Passable  **Description du poste** : CSPS (Maternité)  **Nombre de marraines** : 8 marraines couvrent le CSPS  **Tâche du Poste :**   - Dépistage des grossesses et enregistrement des femmes enceintes, - Programmation et enregistrement des consultations prénatales des femmes enceintes, - Programmation et enregistrement des consultations postnatales des femmes enceintes, - Enregistrement des naissances et suivi des nouveaux nés.   **Les difficultés liées au logiciel** :   - Pas de chronologie de validation des Vat (possibilité de validé Vat5 ensuite Vat 3 ensuite Vat 1), - Le formatage de certains champs pose un problèmed’uniformité.   **Les difficultés Organisationnelles :**   - L’indisponibilité des ordinateurs due aux mises à jour et à la récupération des données qui dure plusieurs jours, ralentie énormément le travail, - Les ordinateurs reviennent souvent avec des incohérences (des pertes de données, des problèmes d’authentification), - Les marraines changent la disposition des puces des téléphones occasionnant des difficultés d’appels, - Les marraines se déplacent souvent sans leurs téléphones.   **Les équipements :**   - Un ordinateur portable HP Probook 4540s, - Deux accumulateurs d’énergie de 100A/H et un convertisseur de tension (24 V), - Une plaque solaire de 250 watts. | |

##

## CSPS de LABARANI

| **Organisme** : Projet MOS@N | |
| --- | --- |
| **Code**: 04  **Date**: 01/05/2015 | Interviewé : NEBILMA BADO  HIEN w Angélique |
|  |  |
| **Localité** : LABARANI  **Niveau de la maitrise du logiciel** : bonne  **Description du poste** : CSPS (Maternité).  **Nombre de marraines** : 8 marraines couvrent le CSPS.  **Attributions du Poste :**   - Dépistage des grossesses et enregistrement des femmes enceintes, - Programmation et enregistrement des consultations prénatales des femmes enceintes, - Programmation et enregistrement des consultations postnatales des femmes enceintes, - Enregistrement des naissances et suivi des nouveaux nés.   **Les Difficultés liées au logiciel** :   - Les paramètres de connexion à la base de données changent souvent de retour de Nouna, - L’impossibilité de modifier l’enregistrementd’une femme enceinte, - Absence de message de confirmation d’ajout ou de modification de femmes enceintes.   **Les difficultés Organisationnelles :**   - L’indisponibilité des ordinateurs pour les mises à jour et la récupération des données au niveau de Nouna, - Le découpage administratif pose un problème d’affection des patientes.   **Les équipements :**   - Un ordinateur portable HP Probook 4540s, - Deux accumulateurs d’énergie de 100A/Het un convertisseur de tension (24 V), - Une plaque solaire de 250 Watts. | |

##

## CSPS de MOURDIE

| **Organisme** : Projet MOS@N | |
| --- | --- |
| **Code** : 05  **Date**: 02/04/2015 | Interviewé : Atounhouyi Nestor |
|  |  |
| **Localité** : Mourdié  **Niveau de la maitrise du logiciel** : bonne maitrise  **Description du poste** : CSPS (Maternité)  **Nombre de marraines** : 7 marraines couvrent le CSPS  **Attributions du Poste :**   - Consultation prénatale, - Consultation postnatale.   **Les Difficultés liées au logiciel** :   - Les paramètres de connexion à la base de données change de retour de Nouna - Impossibilités de modifier une femme enceinte, - Pas de message de confirmation d’ajout ou de modification de femmes enceintes, - Certains formats d’écritures des champs ne sont pas harmonisés.   **Les difficultés Organisationnelles**   - Les Ordinateurs sont souvent indisponibles causé par les mises à jour et récupération des données au niveau de Nouna, - Le découpage administratif pose un problème d’affection des patientes.   **Les équipements :**   - Ordinateur portable HP Probook 4540s, - Deux accumulateurs d’énergie de 100A/H et un convertisseur de tension (24 V), - Une plaque solaire de 250 Watts. | |

## CSPS de SOLIMANA

| **Organisme** : Projet MOS@N | |
| --- | --- |
| **Code** : 06  **Date**: 03/05/2015 | Interviewé : Ouédraogo Lizéta |
|  |  |
| **Localité** : SOLIMANA  **Niveau de la maitrise du logiciel** : bonne  **Description du poste** : CSPS (Maternité)  **Nombre de marraines** : 9 marraines dans les différents villages du CSPS  **Attributions du Poste :**   - Consultation prénatale - Consultation postnatale   **Les Difficultés liés au logiciel** :   - Les messages d’erreur sont en Anglais, - Impossibilités de modifier une femme enceinte, - Pas de message de confirmation d’Ajout ou de modification de femmes enceintes, - Certains formats d’écritures des champs ne sont pas harmonisés (Âge, Poids, Bdc,Hu).   **Les difficultés Organisationnelles**   - Les Ordinateurs sont indisponibles tantôt avec le major, souvent à Nouna pour les Mises à jour, - La lourdeur du travail administratif (Beaucoup de fiches à remplir en plus des actes médicaux) fait que les saisies sur les ordinateurs sont effectuées à la pause.   **Les équipements :**   - Un ordinateur portable HP Probook 4540s, - Deux accumulateurs d’énergie de 100A/H et un convertisseur de tension (24 V), - Une plaque solaire de 250 Watts. | |

# Etude et évaluation de la plateforme technologique

## Etat des équipements

Nous avons répertorié les équipements qui interagissent dans la plateforme technologique du projet MOS@N. Ils sont regroupés en deux groupes à savoir ceux de la salle serveur et ceux installés dans les CSPS.

|  | Les équipements | Quantité |
| --- | --- | --- |
| **Les équipements de la salle serveur** | Serveur HP ProLiant DL320e Gen 8 v2 | 1 |
|  | Router Cisco 2800series | 1 |
|  | Passerelle SMS Hypermédia | 1 |
|  | Serveur Téléphonique YeaStar U100 (PABX) | 1 |
|  | Passerelle Voix Néogate TG 800 ( ) | 1 |
|  | Armoire | 1 |
| **Kit des CSPS** | Ordinateurs portables HP Probook 4540s | 6 |
|  | Téléphones portables des marraines et animateurs | 62 |
|  | Les Plaques Solaires de 250 Watts | 5 |
|  | Les Batteries des 100VA et 12V | 10 |

##
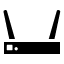

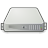
Architecture plateforme

**Serveur téléphonique**

Passerelle VOIX


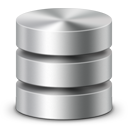


Ecoute des messages de sensibilisation dans les langues de la localité

Bd **PV/VIH**


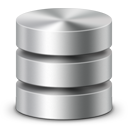


Bd **DARA**


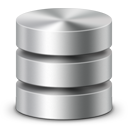

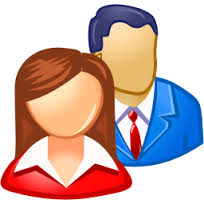


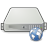

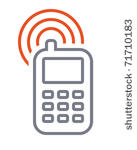


Serveur Web Central

Bd **SIKORO**


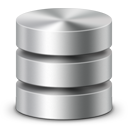


Bd **MOURDIE**

**Marraines ou Animateurs**


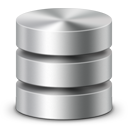


Bd **SOLIMANA**


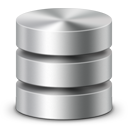


Synchronisation automatique

des BACKUP


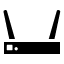


Bd **LABARANI**


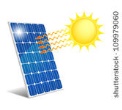


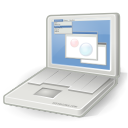


Passerelle SMS


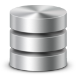

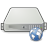


Bd Local

Serveur Web Local

Kit CSPS

Lors des visites des CSPS nous avons testés à chaque étape les connexions entre les équipements ce qui nous a permis de relever des acquis (représenter par les flèches en bleu).

La seule difficulté majeure rencontrée se situe au niveau de la **synchronisation des backups des Bases de données locales vers le serveur**. Cela est dû en grande partie à l’absence ou la mauvaise connexion Internet (3G) de l’opérateur Telmob dans la Zone de Nouna.

## **Sécurité logique et physique**

Les équipements du projet MOS@N sont dans une salle serveur abritant d’autres équipements du Centre. Afin d’assurer la sécurité logique et physique de la plateforme, les dispositions suivantes ont été prises:

- Activation Firewall (flux entrant, flux sortant),
- Cryptage de données sur 128 bytes lors des synchronisations des données,
- Limitation des droits d’accès par la création des comptes utilisateurs simples,
- L’installation des Antivirus sur les postes utilisateurs (dans les CSPS),
- Programmation automatique des Backup des bases locales afin d’assurer une intégrité des données.

Les mesures de sécurité citées devraient découler d’une politique de sécurité bien élaborée et suivie régulièrement. La démarche de réalisation de cette politique est basée sur une[analyse des risques](http://fr.wikipedia.org/wiki/Analyse_des_risques) en matière de sécurité à tous les niveaux.

Aussi nous avons constaté que la sécurité physique de la salle serveur est inappropriée. Ainsi il serait judicieux de conduire un audit de sécurité afin de sécurisé adéquatement les équipements qui s’y trouve.

## Etude du logiciel MobileSanté

### Cartographie des principales fonctionnalités

Le logiciel Mobile Santé a été développé pour la gestion et le suivi des femmes enceintes et patients atteint du VIH. Il regroupe les fonctionnalités suivantes :

- L’enregistrement et la modification des femmes enceintes avec tous les renseignements nécessaires,
- L’enregistrement et la modification des consultations prénatales des femmes enregistrées,
- La Validation des vaccinations VAT des femmes enregistrées,
- L’enregistrement et la modification les accouchements,
- Le suivi des vaccinations des nouveaux nés issues des accouchements,
- L’enregistrement des patients atteints du VIH,
- L’enregistrement des consultations et examens biologiques des patients atteints du VIH,
- L’enregistrement des programmes de vaccination des CSPS,
- Permet la communication de la base de données avec le système automatique de rappel téléphonique des consultations et vaccinations programméesaux marraines et aux animateurs des villages. Ils reçoivent un appel de rappel sur leurs téléphones les 5, 3, 1 avant le jour de la consultation,
- L’administration des utilisateurs et le paramétrage de certaines informations générales tels que les vaccins, les méthodes contraceptives, etc.

### Architecture et technologie de développement Utilisées

**Le progiciel MobileSanté** est développé en PHP avec le Framework Symphony. Elle à une architecture 3tiers et utilise le MVC comme modèle de développement.

**La Base de données** de l’application utilise le SGBD MySQL qui est libre et que nous jugeons performant et pratique pour ce type d’application.

**Apache** est le serveur web qui permet la fluidité des échanges et le traitement des données entre les postes clients et la Base de données (BD).


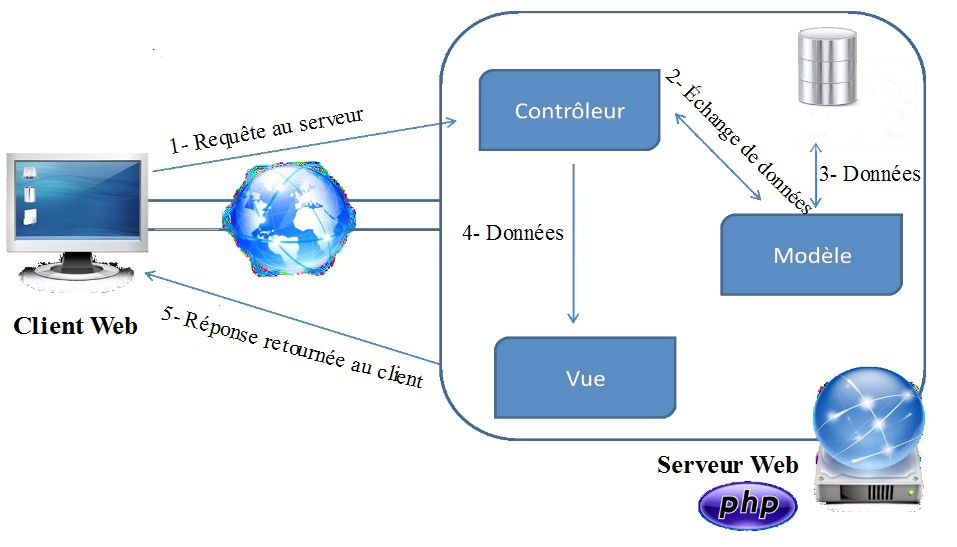


### Evaluation de la Base de données

#### La liste des tables

La base de données de l’application est composée de 40 tables qui sont :

| **N°** | **Nom de la Table** | **Description** |
| --- | --- | --- |
| 1 | Albumine | le taux d’albumine |
| 2 | Alerte | la table des alertes à effectuer |
| 3 | Alertevoix | les alertes voix à déclencher |
| 4 | Animateur | les animateurs du projet mos@n |
| 5 | Association | L’association |
| 6 | Cas | Les cas |
| 7 | Chr | Stocke les centres hospitaliers régionaux |
| 8 | consultationfemme | table qui stoke les consultations des femmes |
| 9 | consultationpvvih | stocke les consultations des PV/VIH |
| 10 | CSPS | stocke les csps du projet |
| 11 | District | renferme les districts du système |
| 12 | Examenbio | renferme les examens biologiques |
| 13 | fos_group |  |
| 14 | fos_user | table des groupes d’utilisateur |
| 15 | Grossesse | table qui stocke les grossesses |
| 16 | Issuegrossesse | stokes les issue de grossesses de façon générale |
| 17 | Langue | stocke les langues parlées |
| 18 | Maladie | stocke maladies du système |
| 19 | Methodecontra | la table des méthodes contraceptives |
| 20 | Paramheurealerte | stocke les paramètres des alertes heures |
| 21 | Paramjouralerte | stocke les paramètresdes alertes jours |
| 22 | Passage | la table des passages |
| 23 | Pays | stocke les pays du projet |
| 24 | Personne | stocke les personnes intervenant dans le projet |
| 25 | Poidsenfant | stocke les poids des enfants |
| 26 | Porteentree |  |
| 27 | Progvaccin | Stocke les vaccins programmés |
| 28 | Reference | Stocke les références |
| 29 | Referencetype | stocke les types de référence |
| 30 | Residence | stocke la résidence |
| 31 | Sensibilvoix |  |
| 32 | Sexe | stocke les genres |
| 33 | Stadeoms | stocke les stades des malades du VIH |
| 34 | Statuttherapeut | stocke les statuts thérapeutiques |
| 35 | Typeaccouchement | stocke les types d’accouchement |
| 36 | Typemalade | stocke les types de malade |
| 37 | Typevirus | stocke les types de virus |
| 38 | Vaccin | stocke les vaccins du district à réaliser |
| 39 | vaccinfemme | renferme les vaccins des femmes à réaliser |
| 40 | village | renferme les villages du projet |

#### Description du modèle Physique de données

Nous avons subdivisé le modèle physique de données suivant les principaux modules de l’application :

- **Module de paramétrage**

- **Module de gestion de femmes enceintes**

- **Module de gestion des patients PV/PIH**

### Les dysfonctionnements de MobileSanté

La synthèse des interviews fait ressortir clairement les difficultés, les bugs et dysfonctionnements constatés par les utilisateurs lors des saisies.

| **N°** | **Dysfonctionnements et points d’amélioration** | **Conséquences** |
| --- | --- | --- |
| 1 | Absence de mots de passe pour accéder à la BD | Volatilité des données |
| 2 | Des messages d’erreurs liés au serveur web qui ne démarre pas automatiquement | L’accès à l’application est impossible |
| 3 | Dans le formulaire d’enregistrement, le champ « **Age de la grossesse »** n’est contrôlé ; il doit être compris entre 6 et 45 semaines | Possibilité de saisir des données erronées touchant du coup à la fiabilité des données |
| 4 | Le format de saisie de valeurs de champs diffère suivant l’utilisateur (Exemple : « 10 kg » diffère de « 10KG » diffère de « 10 Kg »). | Conséquence directes sur l’intégrité des données |
| 5 | Un problème de navigation dans l’application : la validation de certains formulaires ne renvoie aucun message. | Duplication des données dans la base de données dues au fait que les utilisateurs ne sont pas renseignés sur les opérations effectuées. |
| 6 | La possibilité de valider VAT 5 sans avoir à valider les VAT 1, VAT2, VAT 3, VAT 4 | Les validations étant irréversibles alors les données, aucune chronologie ne pourrait établie |
| 7 | L’impossibilité de modifier l’enregistrement d’une femme enceinte. | Les données incohérentes ne pourront pas être corrigées |
| 8 | Un simple clic sur les liens supprimer, décès, perdues de vues effectuer des actions directement. | Des actions seront effectuées dans la base de données de façon récurrente et involontaire surtout avec la sensibilité des souris des ordinateurs portables. |

## Les Aspects Organisationnelles

Le facteur humain influence fortement les résultats finaux attendus des projets informatiques. Pour cela nous avons relevé quelques points d’amélioration au niveau organisationnel afin de permettre l’atteinte des objectifs du projet.

- Sur l’effectif total des agents de santé des 5 CSPS, seulement 3 agents de Labarani, Mourdié et Solimana ont une bonne maitrise de l’application.
- La quasi-totalité des agents de santé les plus concernés (les infirmières de la maternité) sauf celle de Solimana ne maitrisent pas l’application.

# Les Orientations et recommandations

## Les Orientations Organisationnelles

Un certain nombre d’orientations et de recommandations ont été identifiées lors des différents entretiens effectués avec les agents des CSPS et des responsables du projet MOS@N, à savoir :

- Rendre disponible les ordinateurs dans les CSPS pour l’enregistrement régulier des patientes,
- Former adéquatement les agents de santé pour une meilleure maitrise des outils.
- Sensibiliser les marraines à garder, et à mieux gérer leurs téléphones.
- Sensibiliser les marraines à l’utilisation régulière des téléphones afin de recevoir tous les rappels,
- Identifier les problèmes de collaboration entre les agents de santé pour un meilleur rendement,
- Identifier les zones de forte connectivité pour effectuer et recevoir les appels,
- Définir un programme de formation et de recyclage pour les informaticiens pour des aspects jugés pertinents.

## Les Orientations Technologiques

### La sécurité

Les éléments de sécurité déjà en place sont appréciables, mais nous proposons ici d’autres composantes très importantes à prendre en compte pour une meilleure sécurisation des équipements.

La sécurité informatique s'explique par les concepts suivants :

- **la confidentialité:** la sécurité informatique doit protéger les données privées ou les données sensibles qui ne doivent en aucun cas arriver sous les yeux ou entre les mains des personnes malveillantes;
- **l'intégrité:** les données se trouvant sur un ordinateur ou traitées par un logiciel doivent être modifiées seulement par des moyens légitimes et pouvant être vérifiés. La modification de ces données n'est permise que par une source autorisée;
- **la disponibilité:** les données et les services offerts par des ordinateurs et des logiciels doivent être disponibles. L'attention doit porter sur de possibles attaques (de type [DoS](http://www.awt.be/web/sec/index.aspx) ou déni de services) externes ou internes qui mettent en danger la disponibilité d'un système;
- **l'authentification:** chaque byte de données utilisé par un système et chaque utilisateur d'un système doit être identifié et authentifié. Cela signifie qu'il faut vérifier:
- que l'utilisateur est celui qu'il prétend être,
- que chaque donnée qui arrive sur un système provient d'une source de confiance;

La démarche de réalisation de cette politique est basée sur une [analyse des risques](http://fr.wikipedia.org/wiki/Analyse_des_risques) en matière de sécurité à tous les niveaux.

L’analyse des risques doit couvrir les aspects suivants:

- La sécurité organisationnelle et physique ;
- Les bases de données ;
- Les serveurs ;
- Les équipements réseaux LAN et WAN.

Sous réserve d’un audit de sécurité plus approfondie, nos recommandations se porteront sur la sécurité Organisationnelle et physique.

La sécurité physique est le premier rempart (après l’humain) pour assurer la protection des données de l’entreprise, avant même la sécurité logicielle, c’est pour cela que cette dernière ne doit pas être prise à la légère.

La sécurité physique et environnementale vise à empêcher l’accès non autorisé ainsi que les dommages et perturbations de tous genres pouvant affecter les activités quotidiennes et les éléments de l’actif informationnel dans le cadre d’une salle informatique.

Nous préconisons les quelques recommandations qui suivent, classées par risques :

- **Les Risques liés aux incendies**
- L’installation de détecteurs de fumées, de chaleur, flamme,
- L’installation d’extincteur en proximité et à l’intérieur de la salle serveur. Pour une salle contenant du matériel informatique, il est nécessaire d’utiliser un extincteur de catégorie D, les autres pourraient aussi stopper le feu mais en provoquant des dommages sur le matériel informatique et électrique irréparables.
- **Les Risques liés à l’eau ou à une inondation**
- Toutes les prises électriques doivent se situer en hauteur afin d’éviter tout problèmes. De plus, tous les câbles d’une salle serveur doivent passer dans un faux plancher afin de diminuer encore le risque de contact avec une source d’eau,
- Afin de palier efficacement aux surtensions, il existe deux phénomènes naturels à prendre en compte le tonnerre, la foudre en installant un paratonnerre et un parafoudre.
- **Les Risques liés à l’accès à la salle serveur.**
- Renforcer les portes d’accès à la salle serveur c'est-à-dire remplacer ceux existants en bois par des portes métalliques d’épaisseur acceptable qui sont plus adaptés à la salle serveur,
- Afin de restreindre efficacement l’accès à la salle serveur, il y a la solution des clés ainsi que des badges. Seul les personnes de confiance et les personnes ayant obligation d’avoir les droits d’accès doivent les avoir.

### L’architecture Technique de la plateforme

#### Scenario 1


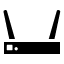

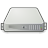
L’architecture demeure la même. L’objectif de ce scénario répond à une problématique liée à la connectivité 3G de l’opérateur TELMOB dans la zone d’action du projet MOS@N. Les Transferts de données se feront manuellement pour le moment afin de permettre la mise en route des fonctionnalités le rappel des rendez-vous des patients. La récupération des données pourra se faire tous les 15 jours afin de permettre les programmations sur le serveur.

Serveur

Téléphonique

Passerelle VOIX


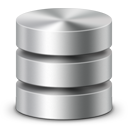


Bd **PV/VIH**


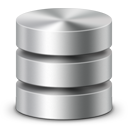


Bd **DARA**


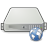

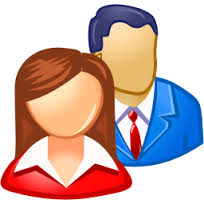

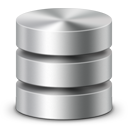


Serveur Web Central


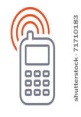


Bd **SIKORO**


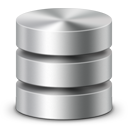


Marraines ou Animateurs

Transfert de sauvegarde

Manuellement

Bd **MOURDIE**


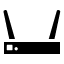

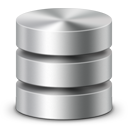


Bd **SOLIMANA**


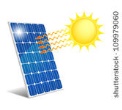

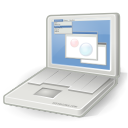

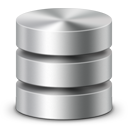


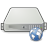

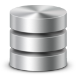


Serveur Web

Local

Kit CSPS

Bd **LABARANI**

Bd Locale

Passerelle SMS

#### Scenario 2

Il se resume à la création d’une base de donnée centrale auquelle les CSPS auront un accès directe via un réseau spécialisé ou Internet (APN TELMOB) . Les données et les traitements se feront en temps réels. La mise en œuvre pratique de ce scénario nécessite une bonne connectivité . Il pourra être exploité si APN de l’opérateur TELMOB sera fonctionnel.


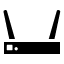

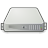


Serveur

Téléphonique

Echange

de données

Passerelle VOIX


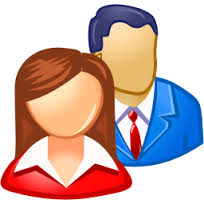


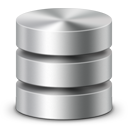

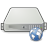

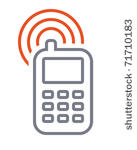


Serveur Web Central

Marraines ou Animateurs

Bd Centrale

Echange de données

via ligne dédiée


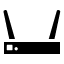


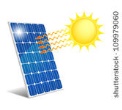


Passerelle SMS


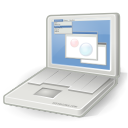


Kit CSPS

#### Synthèse des scénarii

Les deux scénarii proposés pour l’amélioration de l’architecture technique s’inscrivent dans les objectifs du projet.

| Critères | Scénario 1 | Scénario 2 |
| --- | --- | --- |
| Equipements et Ressources requis | Une personne chargé de collecter les backups dans les CSPS chaque 15 jours. | Néant |
| Sécurité | Chaque Base renferme des données partielles .En cela des problèmes d’intégrité de données peuvent être remarqués. | Assure l’intégrité des données dans la base de données centralisée. |
| Administration et déploiement | Données Centralisées. |  |
| Environnement (OS) utilisé  Et logiciel applicatif | Ubuntu et logiciel libre. | Ubuntu et logiciel libre. |
| Accès aux données / réseaux de communication | Le transfert des backups ne nécessite pas obligatoirement de connexion. Il peut se faire manuellement. | Connexion directe au serveur  Via le réseau Internet ou Une ligne dédiée (APN Telmob). |
| Réseau de Communication | Le transfert des backups ne nécessite pas obligatoirement de connexion. |  |
| Maintenance et prévisions futures | Création de nouvelles bases de données pour les CSPS à intégrer dans le futur. Une mise à jour de la structure de la BDdoit s’effectuer sur toutes les BD locales. | Maintenance très facile. |

#

# Ecran de capture de la plateforme


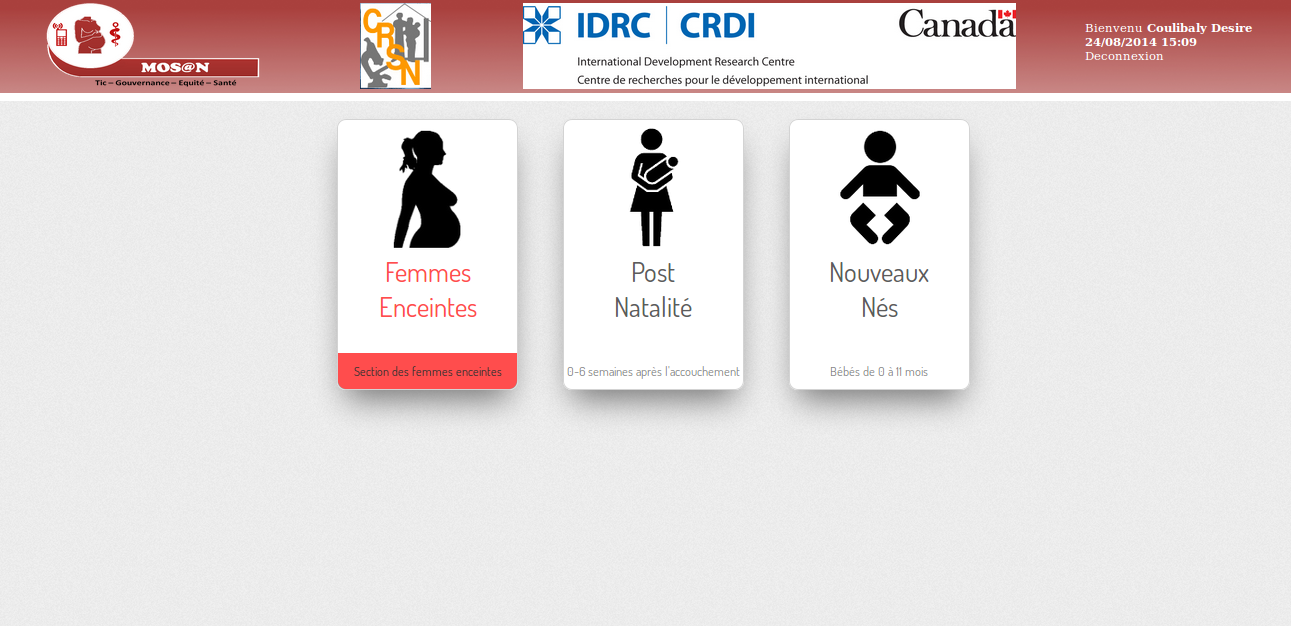


Figure 1 : Ecran d’accueil du module sante maternelle et infantile


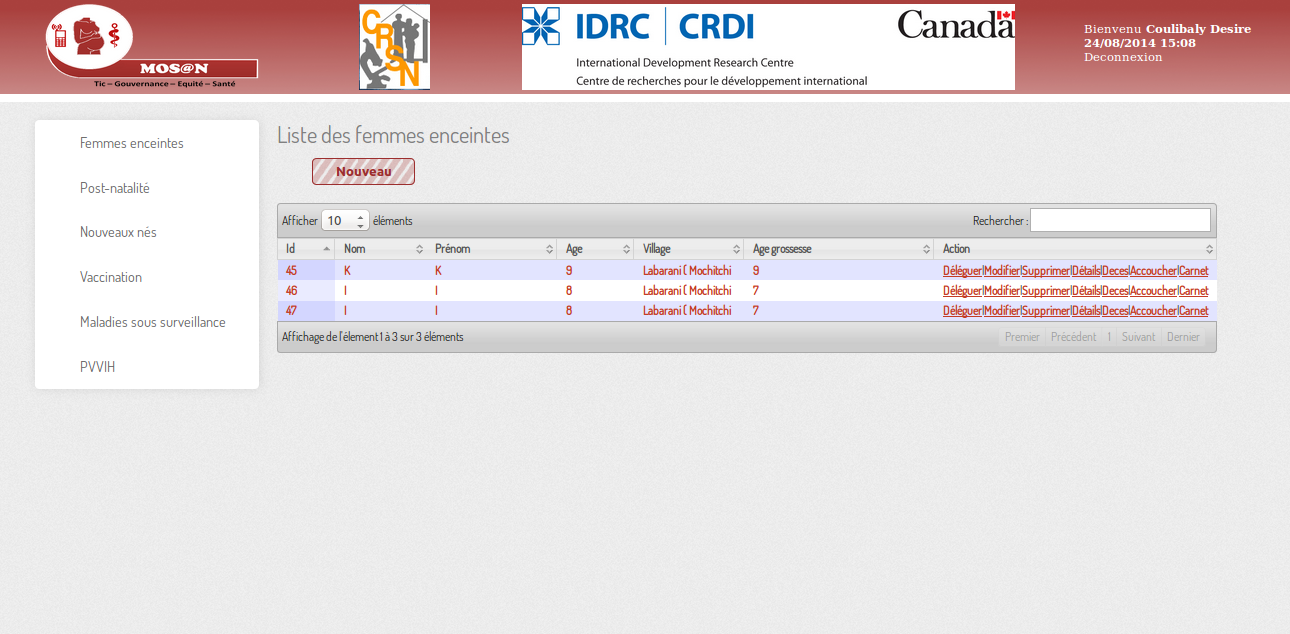


Figure 2 : Page d’accueil création de nouveau client femme enceinte


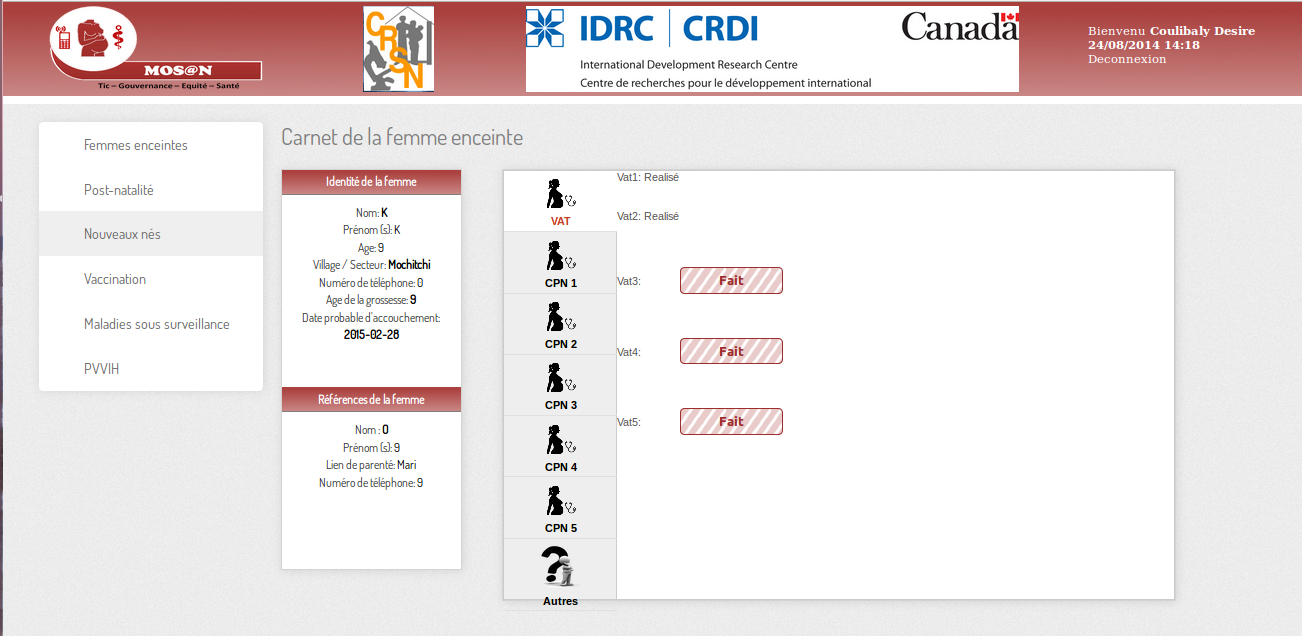


# Figure 3 : Module suivi femme en consultation prénatale

#
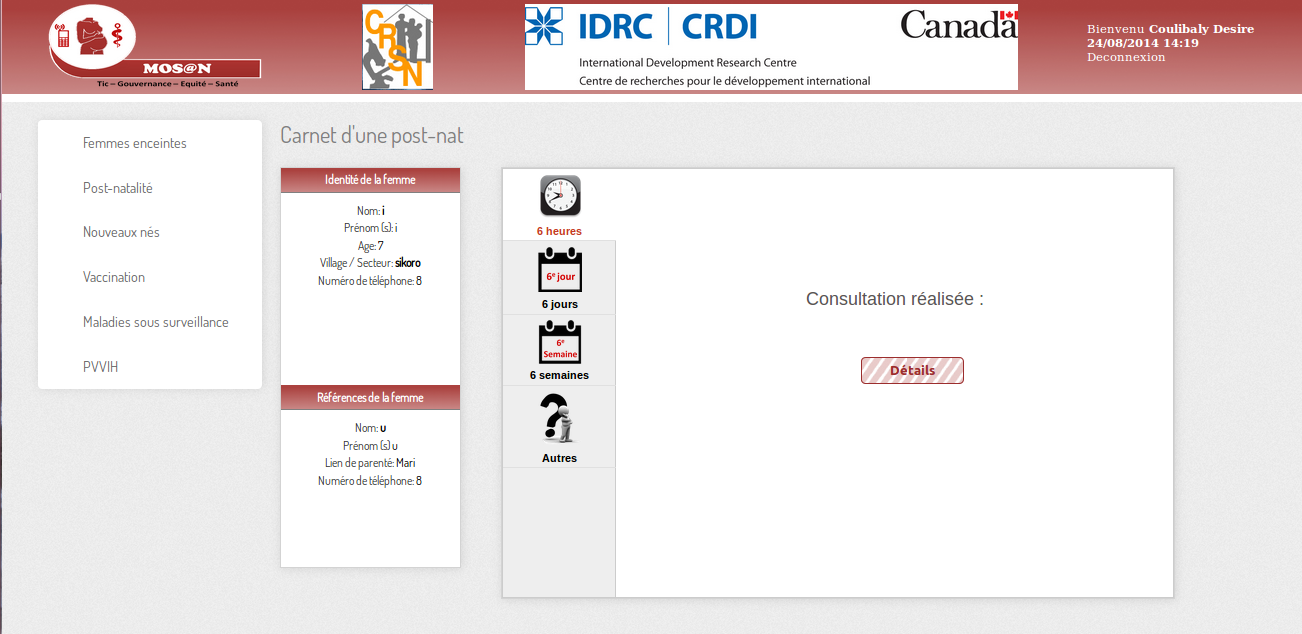


Figure 4 : Module suivi des femmes en consultation post-natale


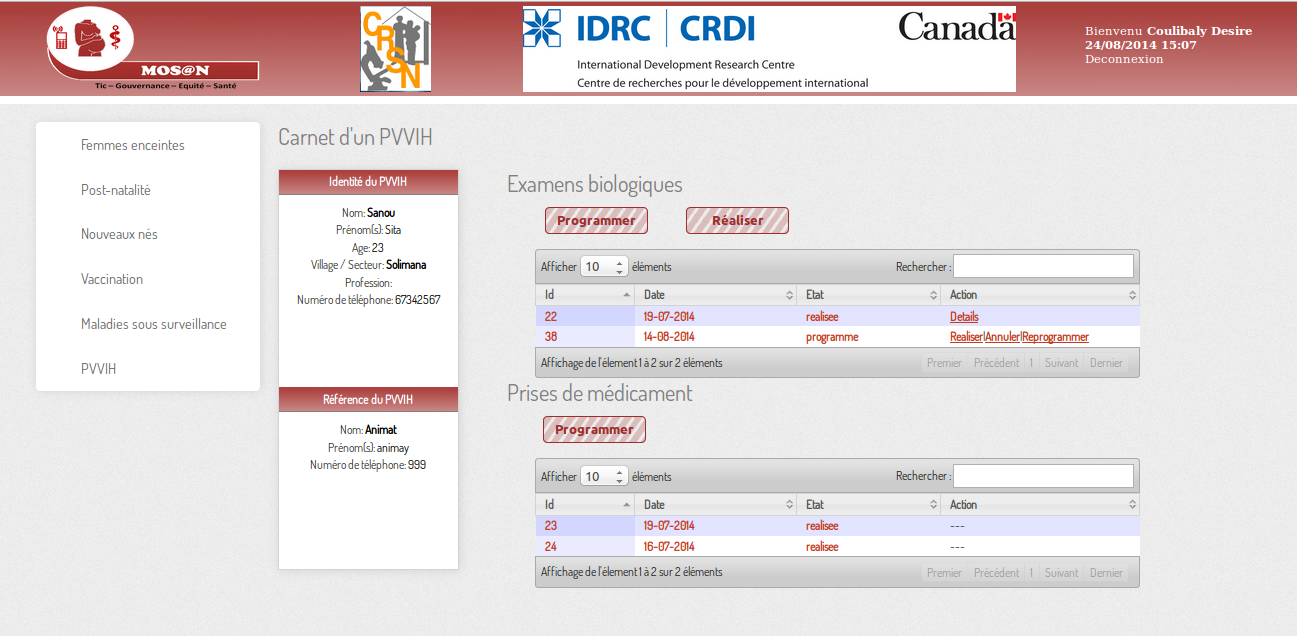


Figure 5 : Module de suivi des PVVIH


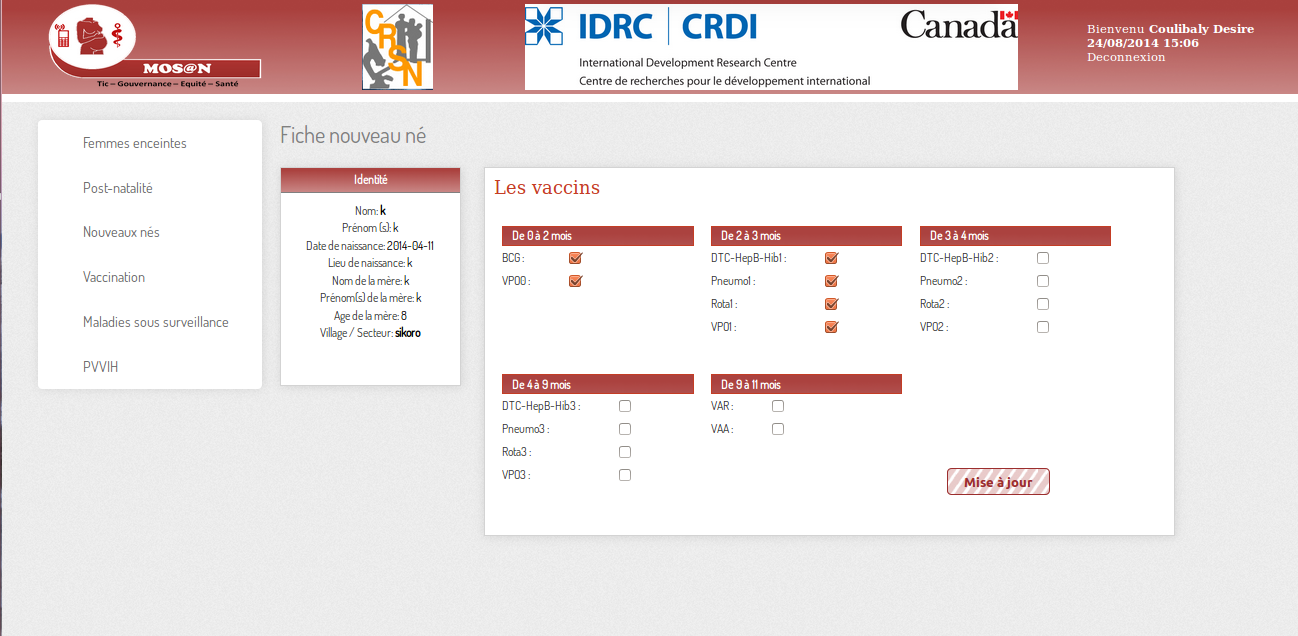


Figure 6 : Module de suivi de la vaccination des moins de 5 ans

# Conclusion

La présente évaluation nous a amené à déceler les difficultés rencontrées sur la plateforme technologique de MOS@N et à faire des recommandations qui seront nécessaires pour que les objectifs nobles du projet soient atteints.

La phase de mise en production de la plateforme est la plus critique et tous les acteurs doivent joués leur partition dans la mise en œuvre des recommandations afin de sécuriser les investissements et de voir des résultats escomptés.
